# Active Learning in Research Methods Classes Is Associated with Higher Knowledge and Confidence, Though not Evaluations or Satisfaction

**DOI:** 10.3389/fpsyg.2016.00279

**Published:** 2016-03-01

**Authors:** Peter J. Allen, Frank D. Baughman

**Affiliations:** School of Psychology and Speech Pathology, Curtin University, PerthWA, Australia

**Keywords:** active learning, research methods, statistics, computer based experiments, authentic data, canned data

## Abstract

Research methods and statistics are regarded as difficult subjects to teach, fueling investigations into techniques that increase student engagement. Students enjoy active learning opportunities like hands-on demonstrations, authentic research participation, and working with real data. However, enhanced enjoyment does not always correspond with enhanced learning and performance. In this study, we developed a workshop activity in which students participated in a computer-based experiment and used class-generated data to run a range of statistical procedures. To enable evaluation, we developed a parallel, didactic/canned workshop, which was identical to the activity-based version, except that students were told about the experiment and used a pre-existing/canned dataset to perform their analyses. Tutorial groups were randomized to one of the two workshop versions, and 39 students completed a post-workshop evaluation questionnaire. A series of generalized linear mixed models suggested that, compared to the students in the didactic/canned condition, students exposed to the activity-based workshop displayed significantly greater knowledge of the methodological and statistical issues addressed in class, and were more confident about their ability to use this knowledge in the future. However, overall evaluations and satisfaction between the two groups were not reliably different. Implications of these findings and suggestions for future research are discussed.

## Introduction

A cornerstone of educational practice is the notion that the more engaged the learner, the more interested, passionate and motivated they will become, and the better the outcome will typically be vis-à-vis their learning. This causal chain, of sorts, thus predicts that higher rates of student retention, better grades, and higher levels of satisfaction and enjoyment are more likely to follow when a student is genuinely curious and involved in their study. However, student engagement appears to be more difficult to achieve in some areas of study compared to others. For instance, within psychology, research methods and statistics are widely regarded as ‘difficult’ subjects to teach (e.g., Conners et al., 1998). Student attitudes toward these topics are often negative (Murtonen, 2005; Sizemore and Lewandowski, 2009), and their interest in them is low (Vittengl et al., 2004; Rottinghaus et al., 2006). This lack of engagement is likely to impact student outcomes, contributing to poorer grades and higher rates of attrition. However, a basic understanding of research methods is essential in order for students to gain a fuller appreciation of the literature underpinning their later academic, or professional careers. Thus, there appears to be a clear and growing need to identify teaching strategies that are maximally effective at removing barriers to learning research methods. This view is echoed by recent calls to reform traditional methods for teaching research methods and statistics, and it finds support from recent research. For example, in the *Guidelines for Assessment and Instruction in Statistics Education* (GAISE; Aliaga et al., 2005) college report, published by the American Statistical Association, a number of recommendations are highlighted with regard to the teaching of statistics in higher education. These recommendations include emphasizing the development of statistical literacy and thinking, making use of real data, focusing on conceptual understanding (rather than procedures or formulae), promoting active learning, making use of technology and administering assessment appropriate to evaluating learning in the classroom.

The view that teaching research methods and statistics may require a particular kind of approach is further supported by a recent meta-analysis by Freeman et al. (2014). In their analysis, traditional methods of teaching statistics (e.g., lecturing to classes) was shown to be less effective in terms of student exam performance, and student satisfaction and enjoyment, compared to other subjects of study. The challenge facing teachers of statistics and research methods therefore is to make research methods more applied, relevant and engaging for students, whilst simultaneously improving students’ understanding of statistics, their grades, and attendance rates (Hogg, 1991; Lovett and Greenhouse, 2000). In this article, we focus on the possible benefits of implementing two of the recommendations highlighted in the GAISE report. These are: (1) the use of real data, and (2) the use of an active learning methodology. We describe a study that examines the ways in which incorporating these recommendations into the teaching of research methods and statistics may positively affect student outcomes.

When applied to the teaching of research methods, active learning approaches typically involve students carrying out research, rather than merely reading about, or listening to instructors talk about it. Active learning in research methods and statistics classes may include taking part in demonstrations designed to illustrate methodological and statistical concepts, participating in authentic research, and working with data the students have been responsible for collecting. A great deal of work has explored the impact of active learning using ‘hands-on’ demonstrations of both statistical processes (e.g., Riniolo and Schmidt, 1999; Sciutto, 2000; Christopher and Marek, 2002; Fisher and Richards, 2004) and methodological concepts (e.g., Renner, 2004; Eschman et al., 2005; Madson, 2005). Importantly, the use of active learning methods in research methods and statistics appears to be successful at increasing levels of satisfaction and enjoyment and reducing failure rates (Freeman et al., 2014). Against this backdrop of findings, it might then seem reasonable to assume that the effects of active learning would further contribute toward positive outcomes, for example on exam performance. However, this is not found to be the case. While students may report higher levels of enjoyment and usefulness of active learning demonstrations, these are not consistently associated with more beneficial learning outcomes (Elliott et al., 2010, though see also Owen and Siakaluk, 2011). Put another way, the subjective evaluation of one’s enjoyment of a subject does not bear a direct relationship on the amount of knowledge acquired, or the extent to which one can apply knowledge in a given area (see e.g., Christopher and Marek, 2002; Copeland et al., 2010).

With regard to the use of real datasets in class exercises and assessments, this too has been proposed to hold a number of advantages (Aliaga et al., 2005). The advantages include: increased student interest; the opportunity for students to learn about the relationships between research design, variables, hypotheses, and data collection; the ability for students to use substantive features of the data set (e.g., the combination of variables measured, or the research question being addressed) as a mnemonic device to aid later recall of particular statistical techniques; and the added benefit that using real data can provide opportunities for learning about interesting psychological phenomena, as well as how statistics should be calculated and interpreted (Singer and Willett, 1990). Additionally, a number of studies have showed that when real, class-generated data are used students report higher levels of enjoyment, an enhanced understanding of key concepts, and are likely to endorse the use of real data in future classes (see e.g., Lutsky, 1986; Stedman, 1993; Thompson, 1994; Chapdelaine and Chapman, 1999; Lipsitz, 2000; Ragozzine, 2002; Hamilton and Geraci, 2004; Marek et al., 2004; Morgan, 2009; Neumann et al., 2010, 2013).

Overall, the benefits of using active learning and real data within research methods and statistics classes show much promise. However, to better understand how the implementation of these strategies results in positive outcomes, further empirical investigation is needed. First, we note a lack of research that has simultaneously targeted outcomes of satisfaction, evaluation and knowledge (i.e., performance). Each of these outcomes likely plays an important role in influencing student engagement. In this study we assess students on each of these components. Secondly, we eliminate a potential design confound that may have affected previous research, by ensuring highly similar contexts in both our intervention and our control group. The same instructors were used in both instances. In this way, we may be more confident that any effects we observe are more likely due to our manipulation (i.e., active learning versus control), than to student-instructor interactions.

Motivated by a desire to increase student engagement in our undergraduate statistics and research methods courses, we developed a series of activities for a 1.5-h workshop. In each of these activities, students participated in a computer-based psychological experiment, engaged in class discussions and activities around the methods used in the experiment, and then used data generated by the class to run a range of data handling and statistical procedures. In this paper, we describe an evaluation of the first of these workshop activities in terms of (a) its subjective appeal to students; and, (b) its pedagogic effectiveness. It was hypothesized that, compared to control participants who were provided with the same content, but delivered using a didactic presentation and canned dataset, students who participated in the activity-based (active learning + real data) workshop would (H1) evaluate the workshop more favorably; (H2) report higher levels of satisfaction with the workshop; (H3) achieve higher scores on a short multiple-choice quiz assessing their knowledge of key learning concepts addressed in the workshop; and (H4) report significantly higher confidence about their ability to demonstrate skills and knowledge acquired and practiced in the workshop.

## Materials and Methods

### Design

A non-equivalent groups (quasi-experimental) design was employed in this study, with intact tutorial classes randomly assigned to the two workshop versions. These workshop versions were equivalent in content, but differed in delivery format. The activity-based version of the workshop began with a computer-based experiment in which the students participated, and contained activities that required students to analyze data collected in class. The canned dataset version of the workshop differed in that it began with a short description of the computer-based experiment (presented by the same instructors as the activity-based workshop), but was otherwise equivalent to the activity-based workshop. As much as possible, the workshops were identical in all other respects. The independent variable in this study was workshop type, of which there were two levels: activity-based and didactic/canned. The four dependent variables were: (1) evaluations, (2) overall satisfaction, (3) knowledge, and (4) confidence.

### Participants

Participants were recruited from a participant pool, within which students are required to participate in at least 10 points worth of research during each semester (or complete an alternate written activity). One point was awarded for participating in the current study. A total of 39 participants were obtained for final analysis. Initial comparisons between the activity-based group (*n* = 25; *M* age = 22.43, *SD* = 4.95; 68% female; *M* final grade = 61.12, *SD* = 14.54) and the didactic/canned group (*n* = 14; *M* age = 25.93, *SD* age = 12.27; 78.6% female; *M* final grade = 61.42, *SD* = 11.90) indicated that there were no reliable group differences in age, *t*(15.59) = -1.22, *p* = 0.230, *d* = 0.37, gender distribution, χ^2^ (1, *N* = 39) = 0.50, *p* = 0.482, φ = 0.11, or final semester grades, *t*(36) = -0.066, *p* = 0.948, *d* = 0.02.

This research complies with the guidelines for the conduct of research involving human participants, as published by the Australian National Health and Medical Research Council (The National Health, Medical Research Council, the Australian Research Council, and the Australian Vice-Chancellors’ Committee [NH&MRC], 2007). Prior to recruitment of any participants, the study was reviewed and approved by the Human Research Ethics Committee at Curtin University. Consent was indicated by the submission of an online evaluation questionnaire, as described in the participant information immediately preceding it.

### Materials and Measures

#### Workshop

The activity-based version of the workshop commenced with students participating in a short computer based experiment designed to examine the effects of processing depth on recall. Class members were randomized to one of two processing conditions, *imagine* and *rehearse*, then asked to remember a list of 12 words presented on screen at a rate of one word every 2 s. Members of the *imagine* condition were encouraged to engage in deep processing by being instructed to “try to imagine each concept as vividly as possible such that you are able to remember it later.” Members of the *rehearse* condition were encouraged to engage in shallow processing by being instructed to “try to rehearse each word silently such that you are able to remember it later.” All students then completed multiplication problems for 150 s as a distractor task. Finally, all students were presented with 24 words, 12 of which were ‘old’ (i.e., appeared on the original list) and 12 of which were ‘new’. They were asked to indicate whether each of the 24 words was ‘old’ or ‘new’ by pressing a relevant keyboard button.

This task was developed in Java by the second author, as existing commercial software packages were unsuitable for our purposes due to high annual licensing fees (e.g., St James et al., 2005), or an insufficient feature set (e.g., Francis et al., 2008). It was hosted on a private webserver, and accessed by students using a standard web browser (e.g., Firefox). The data generated by each student were saved to a MySQL database accessible to the class tutor from his/her networked workstation. Following their participation, students were provided with a brief written summary of the experiment, and asked to work together to address a series of questions about its key methodological features. These questions prompted students to identify and operationalize independent and dependent variables, write research and null hypotheses, visualize experimental designs using standard notation, and consider the purpose of randomization.

While the students worked on these questions, the tutor downloaded the class data and collated them into an SPSS data file that was subsequently uploaded to a network drive for students to access. After a brief class discussion around the methodology of the experiment, students were directed to open the SPSS data file, and commence work on a series of questions requiring various data handling techniques and statistical analyses to address. Specifically, students were required to identify the appropriate statistical test to compare the two conditions on classification accuracy, and then run, interpret and report (in APA style) an independent samples *t*-test (including assumption testing, and an effect size). The workshop concluded with a class discussion around the statistical analyses, findings and interpretation.

The didactic/canned version of the workshop was identical to the activity-based version, except it began with a short description of the computer based experiment (presented by the class tutor with the aid of PowerPoint slides), and required students to analyze a canned data set, rather than class generated data.

#### Evaluation Questionnaire

The online evaluation questionnaire contained five sections, measuring the four DVs and capturing key demographic data. It is reproduced in full in the Appendix (available as **Supplementary Material Data Sheet 1**).

##### Section 1 (evaluations)

Section 1 of the online questionnaire contained 13 items assessing students’ evaluations of the workshop. Although there are numerous measures that have been developed to allow students to evaluate units and courses, a review of the literature indicated that there are currently no instruments suitable for evaluating specific activities embedded within a unit or course. Consequently, this measure was developed specifically for the purposes of the current research (although inspired by the single-item measures that are frequently used in evaluations of teaching activities reported elsewhere). Participants responded to each item on a 7-point scale ranging from 1 (*Strongly disagree*) to 7 (*Strongly agree*), and examples of items on this measure include “this workshop was useful” and “this workshop was an effective way of teaching research methods and statistics.” Although a small sample size limited our ability to examine the factor structure of this measure (for example, Pett et al. (2003), suggest a minimum of 10–15 cases per item for exploratory factor analysis), Cronbach’s alpha was 0.96, indicating that it was internally consistent. Responses to the 13 items were summed to provide an overall index of how favorably students rated the workshop.

##### Section 2 (satisfaction)

The second section of the online questionnaire was a single item measure of overall satisfaction with the workshop, which respondents answered on a scale ranging from 1 (*Very Dissatisfied*) to 10 (*Very Satisfied*). The correlation between this single item measure and the sum of responses to the 13-item evaluation scale was *r* = 0.91, suggesting that they measured overlapping constructs.

##### Section 3 (knowledge)

Five multiple-choice questions were used to assess knowledge of the key learning outcomes addressed in the workshop. Each question provided four response options, of which only one was correct, thus total scores on this measure ranged from 0 to 5.

##### Section 4 (confidence)

This section of the questionnaire asked respondents to indicate on a 4-point scale ranging from 1 (*Not at all confident*) to 4 (*Very confident*) their confidence regarding their ability to apply seven specific skills developed in the workshop, assuming access to their notes and textbook. For example, “run and interpret and independent samples *t*-test using SPSS.” Again, the small sample size limited our ability to examine the factor structure of this measure, although Cronbach’s alpha was 0.84, indicating that it was internally consistent. Responses to the items on this measure were summed to provide an overall index of student confidence.

##### Section 5 (demographics)

The final section of the evaluation questionnaire asked students to specify their age, gender, and the day/time of the workshop they attended. The day/time information was used to assign participants to the levels of the independent variable.

### Procedure

Before the start of semester, tutorial classes were block-randomized to the two workshop versions. The workshop was then delivered as part of the normal tutorial schedule. Participants were provided with an information sheet outlining the nature of the current study, and it was stressed that their involvement was (a) entirely voluntary, and (b) anonymous to the unit’s teaching staff. At the end of the workshop, students were reminded about the research, and asked to complete the online evaluation questionnaire, which was linked from the unit’s Blackboard site, within 48 h of the class finishing. Prior to accessing the online questionnaire, participants were presented with an online version of the information sheet hosted on our school website, as recommended by Allen and Roberts (2010).

## Results

Each hypothesis was tested with a Generalized Linear Mixed Model (GLMM), implemented via SPSS GENLINMIXED (version 22), with an alpha level of 0.0125 (to protect against the inflated risk of making Type 1 errors when conducting multiple comparisons on a single data set), and robust parameter estimation. GLMM is preferable to a series of independent samples *t*-tests or ordinary least squares (OLS) regression analyses, as it can accommodate dependencies arising from nested data structures (in this instance, 39 students nested in seven classes, facilitated by three tutors), non-normal outcome variables, and small, unequal group sizes. In each GLMM, there were two random effects (class and tutor)^1^ and one fixed effect (condition) specified. A normal probability distribution was assumed for each outcome variable, and each was linked to the fixed effect with an identity function.

The fixed effects from the four GLMMs are summarized in **Table 1**, where it can be seen that members of the activity-based condition scored significantly higher than members of the didactic/canned condition on the knowledge and confidence measures, but not the evaluation and satisfaction measures. When indexed using Hedges’ g, the knowledge and confidence effects could be characterized as ‘large’ and ‘small,’ respectively.

**Table 1:** Summary of differences between the two conditions on the four outcome variables.

Outcome	Estimated condition *M* (*SD*)		95% CI	*t*	*p*	*g*
					
	Activity-based	Didactic/canned				
Evaluations	4.77 (1.22)	4.71 (0.31)	[-0.59,0.72]	0.21	0.836	0.06
Satisfaction	6.68 (2.86)	7.00 (0.95)	[-2.02,1.37]	-0.38	0.703	0.13
Knowledge	3.72 (0.32)	3.43 (0.11)	[0.22,0.36]	8.07	<0.001	1.07
Confidence	2.38 (0.20)	2.33 (0.12)	[0.04,0.08]	5.86	<0.001	0.28


*95% CI = 95% confidence interval of the difference between two means. *g* = Hedges’ *g* for the weighted standardized difference between two means. *N* = 39 for all outcomes except Satisfaction, where *N* = 38.*

## Discussion

We have focused on the implementation of two recommended strategies for teaching research methods and statistics: using real data, and following an active learning approach. Our results showed no reliable differences between groups in their rated evaluation of (H1), or satisfaction with (H2) the workshops. Those participants in the activity-based workshop were statistically no different in their views to those in the didactic/canned workshop. Indeed, it is interesting to note that both groups rated the workshops to be below-average (i.e., below the neutral-point) on the evaluation and satisfaction measures, suggesting that their views regarding the workshops were somewhere between ambivalent and negative. Overall, these findings were not as we predicted. Rather, we expected students in the activity-based workshop to find more satisfaction with their workshop and evaluate their learning experience more favorably. In-line with our predictions, however, was the finding that on the outcome measure of knowledge/performance, the activity-based group did significantly outperform those in the didactic/canned workshop (H3). Thus, while the groups did not differ in their apparent engagement, they nevertheless achieved different levels of knowledge. Also noteworthy, was the finding that the activity-based group were reliably different to the didactic/canned group in their reported levels of confidence to later apply the skills developed in the workshop (H4).

Seemingly, the results of this study sit at odds with the ‘causal chain’ we described in the introduction. One possible explanation is that for student satisfaction to be positively affected, students need to see the results of their engaged learning first, and perhaps these positive attitudes require time to accumulate. In our study, participants did not have this opportunity. A more interesting possibility is that rather than greater engagement being instrumental in promoting greater levels of satisfaction and enjoyment, which in turn promotes learning, that instead, one’s level of satisfaction is in fact rather separate to the process of learning. If so, this would indicate that a combination of teaching strategies is needed to produce positive outcomes and student engagement. Accordingly, our results would be consistent with previous research that suggests exposure to research methods and statistics in an engaging environment can improve students’ knowledge without necessarily affecting their attitudes (e.g., Sizemore and Lewandowski, 2009). This latter interpretation offers up a variety of potentially interesting research avenues. Minimally, the results of this study suggest against the tailoring of content in educational curricular, based on the reported levels of satisfaction of students.

### Limitations

While the results of the current study raise intriguing questions about the relationship between academic outcomes and self-reported student satisfaction and evaluations, it is important to note a number of possible limitations to the approach we took. The first of these concerns the relatively small, unequal number of participants in the activity-based (*n* = 25) versus canned/didactic (*n* = 14) groups. Clearly, to be more confident in our results, this study requires replication with a larger, more evenly spread sample. A second sampling limitation concerns the randomization of intact groups to conditions. Ideally, we would have randomized individual participants to either the activity-based or didactic/canned workshop, allowing for a true experimental test of each hypothesis. However, this was not possible due to the fact that students self-select into classes based on personal preferences and commitments.

A further possible limitation concerns the analytical approach we chose. Had we opted for another approach, for example independent samples *t*-tests, no reliable differences would have emerged (*p*s 0.385–0.839) and the implications of our study would be quite different. However, due to the fact that participants were recruited across a number of tutorial groups (*n* = 7) supervised by a number of instructors (*n* = 3), we deemed the use of GLMM procedures to be most appropriate. This is because GLMM is aptly suited to dealing with hierarchical data, and clustering effects that may have been present within nested groups of tutorials and instructors. GLMM has the further advantage over the *t*-test in that it may be more robust to dealing with unequal sample sizes (Bolker et al., 2009). Although our analysis showed no such clustering effects, in light of the sampling limitations, GLMM remained most suited to the data.

## Conclusion

This paper describes the implementation and quasi-experimental evaluation of a relatively short (1.5 h) class activity in which students participated in an authentic computer-based psychological experiment, engaged in class discussion around its methods, and then used class-generated data to run a ange of data handling procedures and statistical tests. Results indicated that students who participated in this activity scored significantly higher than participants in a parallel didactic/canned class on measures of knowledge and confidence, but not on overall evaluations or satisfaction. In contrast to the view that student satisfaction is paramount in achieving positive learning outcomes, the results of the current study suggest that, at least during some points in the learning process, one’s level of satisfaction has little effect. This would indicate that a combination of teaching strategies is needed to produce both positive outcomes and student engagement. Future research that employs large-scale, fully randomized experimental designs may have the best chance of revealing these strategies (Wilson-Doenges and Gurung, 2013).

## Authors Contributions

PA conceived and designed the study and analyzed the data. PA and FB co-authored this manuscript. FB programmed the experimental task used as one level of the IV, wrote the documentation and spreadsheets used by the tutors to aggregate the data for class use, and contributed to the overall design of the study.

## Conflict of Interest Statement

The authors declare that the research was conducted in the absence of any commercial or financial relationships that could be construed as a potential conflict of interest.

## Footnotes

1 Note that for five of the eight tests of random effects, the variances were negative, and consequently set at zero during analyses. For iterative procedures (e.g., maximum likelihood estimation), this can occur when the variance attributable to a random effect is relatively small, and the random effect is having a negligible impact on outcome of the analyses. The remaining three random effects were non-significant, with Wald’s Z ranging from 0.298 to 0.955 (*p* = 0.765 to 0.340). Despite their non-significance in the current context, the random effects of class and tutor were retained in our analyses, based on the common recommendation that non-independence of observations attributable a study’s design ought to be routinely accounted for, regardless of the estimated magnitude of its impact (Murray and Hannan, 1990; Bolker et al., 2009; Thiele and Markussen, 2012; Barr et al., 2013).
