# Supplementary material for: Active Learning in Research Methods Classes Is Associated with Higher Knowledge and Confidence, Though not Evaluations or Satisfaction
Source: Front Psychol. 2016 Mar 1;7:279. doi: 10.3389/fpsyg.2016.00279 (PMC4771750; doi:10.3389/fpsyg.2016.00279)
Supplement: Supplementary file 1 [file Data_Sheet_1.DOCX]

# Appendix

# Evaluation and satisfaction measures

The following items ask for your impressions of the workshop this week. Please be as candid and truthful as possible, remembering that I cannot identify you based on your responses to this questionnaire.

Respond to each of the following 13 items on the scale ranging from 1 = *Strongly Disagree* to 7 = *Strongly Agree*.

|  | Strongly Disagree | Strongly  Agree |
| --- | --- | --- |
| 1. Overall, I found this workshop interesting. | 1 2 3 4 5 6 7 | |
| 2. The content of this workshop was easy to understand. | 1 2 3 4 5 6 7 | |
| 3. This workshop was a good supplement to the lectures and textbook readings. | 1 2 3 4 5 6 7 | |
| 4. This workshop was useful. | 1 2 3 4 5 6 7 | |
| 5. This workshop helped me to understand material covered in the lectures and textbook readings. | 1 2 3 4 5 6 7 | |
| 6. This workshop activity should be used again in [this course] next year. | 1 2 3 4 5 6 7 | |
| 7. This workshop was an effective way of teaching research methods and statistics. | 1 2 3 4 5 6 7 | |
| 8. This workshop captured and held my interest. | 1 2 3 4 5 6 7 | |
| 9. This workshop was a valuable learning experience. | 1 2 3 4 5 6 7 | |
| 10. This workshop was engaging. | 1 2 3 4 5 6 7 | |
| 11. I have a better understanding of the lecture content and readings after completing this workshop. | 1 2 3 4 5 6 7 | |
| 12. I understood the material covered in this workshop. | 1 2 3 4 5 6 7 | |
| 13. This workshop was well organized. | 1 2 3 4 5 6 7 | |

Finally, on a scale from 1 to 10, please indicate your overall level of satisfaction with this workshop.

Very dissatisfied 1 2 3 4 5 6 7 8 9 10 Very satisfied

# Knowledge measure

The following five questions assess the knowledge and skills that you developed in Workshop 1. Please complete them WITHOUT LOOKING AT YOUR NOTES OR TEXTBOOK.

This is NOT A TEST, and it I will not be (and, in fact, cannot) match up your answers on these questions with your name or student ID number. I only want to get a sense of what you learned, and what you remembered, from the workshop this week.

I will make these questions (along with the answers) available to ALL students after data collection for this phase of the research has finished (i.e., next week).

1. Tom believes that studying while listening to music will improve test performance. To test this hypothesis, Tom randomizes his participants to two groups. The first group of participants listens to music while studying for a spelling test. The second group of participants studies for the test in silence. Tom then compares the spelling test performance for both groups. Tom’s study is best described as:
   1. An independent samples *t*-test.
   2. A post-test only control group design.
   3. A non-equivalent groups design.
   4. A pre-test post-test pre-experiment.
2. In Tom’s study, the IV is ________ and the DV is _________.
   1. Whether or not the participants listen to music while studying; spelling test performance.
   2. Spelling test performance; whether or not the participants listen to music while studying.
   3. Measured; manipulated.
   4. Scale; nominal.
3. What is the null hypothesis for Tom’s study?
   1. The group ‘music’ group will have higher spelling test scores than the ‘silence’ group.
   2. *M*_1_ > *M*_2_.
   3. *M*_1_ - *M*_2_ = 0.
   4. *M*_1_ < *M*_2_.
4. Looking at the output below:


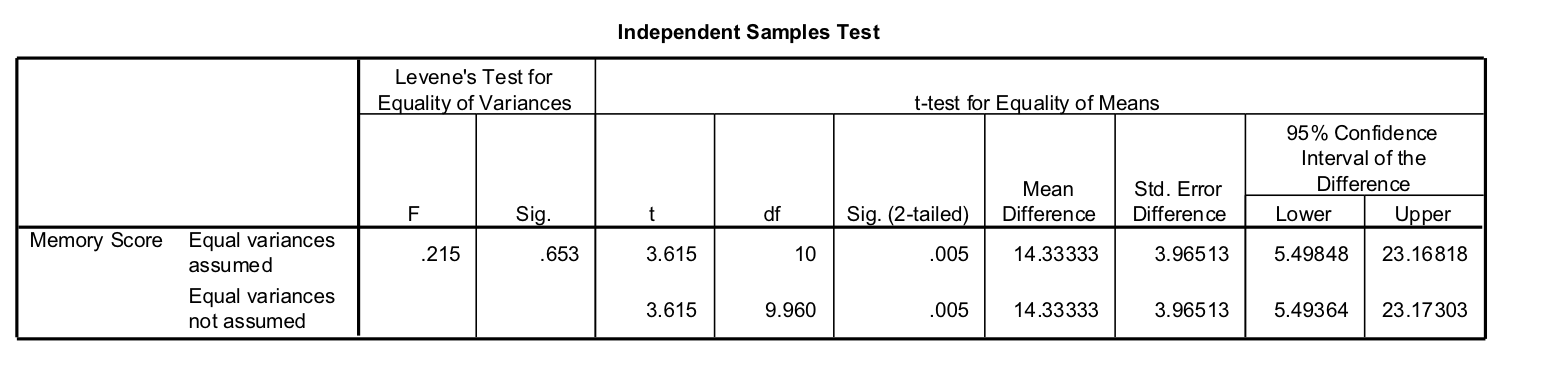


The statistical sentence is:

- 1. *t* (10) = .215, *p* = .653.
  2. *t* (10) = .215, *p* = .005.
  3. *t* (10) = 3.62, *p* = .005.
  4. *t* (9.96) =3.62, *p* = .005.

1. If *p* < .05, what exactly does this tell us?
   1. The probability that the H0 is true is less than .05.
   2. If the H0 is true, the probability that our sample result is due to sampling error alone is less than .05.
   3. If the H0 is true, our sample result is “likely”.
   4. The probability that the research hypothesis is false is less than .05.

# Confidence measure

After Workshop 1, how confident are you that, with your notes and textbooks, you could:

|  | Not at all confident | Somewhat confident | Confident | Very confident |
| --- | --- | --- | --- | --- |
| 1. Correctly identify a post-test only control group design based on a demonstration or description similar to the one at the start of the workshop this week? | 1 | 2 | 3 | 4 |
| 2. Correctly identify the IV and DV in an experiment similar to the one we looked at in the workshop this week? | 1 | 2 | 3 | 4 |
| 3. Write a hypothesis for an experiment based on a demonstration or description similar to the one at the start of the workshop this week? | 1 | 2 | 3 | 4 |
| 4. Correctly set up an SPSS data file for a post-test only control group design? | 1 | 2 | 3 | 4 |
| 5. Test the assumptions of the independent-samples *t*-test using SPSS? | 1 | 2 | 3 | 4 |
| 6. Run and interpret an independent samples *t*-test using SPSS? | 1 | 2 | 3 | 4 |
| 7. Report the results of an experiment similar to the one we looked at in the workshop this week? | 1 | 2 | 3 | 4 |

# Demographics measure

Finally, please answer these four demographic questions.

1. Age _____________

2. Gender ________________

3. Degree:

- Bachelor of Psychology
- BSc (Psych) / BComm (HRM & IR) Double Degree
- Other

4. Workshop that you attended this week:

- Class 1 Monday 18:00 to 19:30
- Class 2 Tuesday 12:00 to 13:30
- Class 3 Tuesday 13:30 to 15:00
- Class 4 Tuesday 15:00 to 16:30
- Class 5 Thursday 12:00 to 13:30
- Class 8 Thursday 13:30 to 15:00
- Class 6 Thursday 15:00 to 16:30

Thank you for taking the time to complete this questionnaire. Please click the “Submit” button to finish.
